# Assessment of Dental Caries among Adolescents in Private Educational Institutions of Kabul City

**DOI:** 10.12688/f1000research.174734.2

**Published:** 2026-07-28

**Authors:** Ahmad Khalid Aalemi, Ali Maisam Eshraqi

**Affiliations:** 1 Department of Oral Medicine, Kabul University of Medical Sciences, Kabul, 1001, Afghanistan; 2 Department of Endodontics and Operative Dentistry, Kabul University of Medical Sciences, Kabul, Afghanistan

**Keywords:** Dental Caries; Prevalence; Adolescent; School going; Kabul

## Abstract

**Objective:**

Dental caries continues to be a significant public health concern, particularly prevalent during adolescence. This study aimed to evaluate the prevalence of dental caries among adolescents aged 10 to 18 years attending two different types of private schools in Kabul City.

**Materials and Methods:**

This descriptive cross-sectional study included 381 school-going adolescents. Data was collected through pre-structured questionnaires and face-to-face interviews, while oral examinations were conducted by an experienced dentist. Both primary and permanent dentitions were assessed for decayed, missing, and filled teeth. Data analysis was performed using SPSS version 21.0, with p < 0.05 considered statistically significant.

**Results:**

The mean age of participants was 14.6 ± 2.13 years, predominantly boys, as girls above grade six are not permitted to attend school in Afghanistan. The prevalence of dental caries was high (70.1%) and decreased with increasing age (p = 0.004), number of siblings (p < 0.001), and better tooth-brushing habits (p = 0.025). The mean DMFT score was 2.40 ± 2.45. Overall, 45.4% of students had good oral hygiene, 11.3% had poor oral hygiene, and 46.7% presented with dental calculus. Both dental calculus and oral hygiene were significantly associated with tooth-brushing habits and use of dental cleansing agents (p < 0.001).

**Conclusion:**

This study highlights a high prevalence of dental caries among students aged 10–18 years at private schools in Kabul, varying by school type. Factors such as age and tooth brushing habits significantly influence dental health, emphasizing the urgent need for targeted oral health education and interventions.

## Introduction

Dental caries is a non-communicable chronic disease that leads to the progressive destruction of tooth structure. ^1^ Despite being easily preventable, it remains a major global public health issue, particularly affecting children and adolescents. ^2^ The distribution of caries is influenced by its etiology. The process begins sub-clinically with the accumulation of cariogenic bacteria in dental plaque, a transparent biofilm that forms on tooth surfaces from birth. These bacteria feed on fermentable carbohydrates, producing acids that dissolve and demineralize the tooth surface. Poor oral hygiene and a diet high in sugar lead to unchecked demineralization, resulting in cavities. If untreated, these cavities progressively damage the tooth structure. ^3^ Regular oral hygiene practices, particularly using fluoridated toothpaste, can significantly reduce the incidence of dental caries. ^4^

In many countries, population-based surveys or surveillance systems to monitor oral health are not prioritized in public health initiatives. ^5^ Afghanistan is among these countries and lacks nationwide epidemiological data on the prevalence of oral diseases, with only a few studies conducted in provinces like Kabul and Herat. A study conducted in Kabul focused on schoolchildren aged 7 to 13 attending government schools in the city. ^6^ Adolescence is a critical period for oral health because dietary habits, oral hygiene behaviors, and independence in self-care become more established during these years, potentially increasing the risk of dental caries. Furthermore, private schools in Kabul represent an important but understudied population with potentially different socioeconomic backgrounds, dietary patterns, and access to oral healthcare compared with students attending public schools. Therefore, assessing dental caries among adolescents in private schools may provide valuable information for understanding oral health patterns in this population. In this study, we aim to assess the prevalence of dental caries among schoolchildren aged 10 to 18 years in two different types of private schools in Kabul City.

## Materials and methods

This descriptive cross-sectional study was conducted on students aged 10 to 18 years in two different types of private schools in Kabul City. One Afghan Turk Maarif school was randomly selected from six eligible Afghan Turk Maarif schools operating in Kabul City. Afghan Turk Maarif Darul Ulum, a full-time residential school funded by the Turkish government and located in the 12th educational district of Kabul, was selected for the study. In addition, one other type of private school was randomly selected from private schools located in the 6th educational district of Kabul City. Shokoh High School, established in 2008, was selected as the second school. This school operates similarly to governmental schools, except that students are required to pay tuition fees.

In each selected school, all students from the selected classes who met the eligibility criteria were invited to participate in the study. Students aged 10–18 years who were present on the day of examination and provided informed consent/assent were included. Students who were absent during data collection or declined participation were excluded. This sampling approach was used to maximize participation and minimize selection bias.

Key variables assessed included age, sex, parental literacy, parental occupation, frequency of tooth brushing, dental floss usage, and oral hygiene practices. In this study, dental carriers were defined as the presence of clinically detectable cavitated lesions on at least one tooth surface. Non-cavitated enamel lesions (white spot lesions) were not considered dental caries for the purpose of this study. Dental caries experience was assessed using the DMFT index, which records the number of decayed (D), missing (M), and filled (F) permanent teeth. Filled teeth were included in the DMFT score because they represent previous caries experience and prior treatment of dental decay. Good oral hygiene was characterized by clean teeth, the absence of visible dental and gingival disease, and no halitosis. Dental calculus was defined as hardened deposits formed by the accumulation and mineralization of bacterial plaque on tooth surfaces. 

Ethical approval for the study was obtained from the institutional review board of Kabul University of Medical Sciences (Reg No: 413/16-09-2018), and the study was conducted in accordance with the principles of the Declaration of Helsinki. Written informed consent was obtained from the parents, and assent was obtained from students before participation. To ensure student safety during oral examinations, single-use instruments and equipment were utilized, and examinations were conducted under strict sanitary conditions.

Data was collected using a pre-structured questionnaire that had been used in our previous study ^6^ and administered through interviews. The oral examinations were carried out by an experienced dentist using dental probes and mouth mirrors under adequate lighting conditions.

Statistical analysis was performed using the Statistical Package for Social Sciences (SPSS) version 21.0. Descriptive characteristics were summarized using means (±SD) and proportions as appropriate. The Pearson Chi-square test was used for comparison of percentages, while Student’s t-test or Mann Whitney U test was applied for comparison of means as appropriate. A p-value of <0.05 was considered statistically significant.

## Results

This study includes 381 participants from two schools, Afghan Turk and Shokoh. The Afghan Turk group is composed exclusively of boys, whereas Shokoh has a more varied distribution with 88.4% boys and 11.6% girls, the latter limited to grade 6 or below. Age demographics reveal that, at the time of the study, Afghan Turk had no participants aged 12 or younger, while 41.4% of Shokoh participants fell into this age category. The largest age group across both schools is 13 to 15 years, representing 44.1% of the total sample. Maternal literacy levels varied between the groups, with approximately one-third (37%) of Afghan Turk participants reporting that their mothers were literate compared with 47% among Shokoh participants, resulting in an overall maternal literacy rate of 41.7%. Paternal literacy is comparatively high in both groups, with 81.4% of fathers being literate. The majority of mothers in both groups are housewives, with a slightly higher percentage in Afghan Turk (91%) than in Shokoh (82.9%). Paternal employment demonstrates diversity, with 37.8% of fathers across both groups employed in jobs that require education, 34.9% in business, and 27.3% in jobs that do not require education. Family size also shows notable differences. Afghan Turk participants tend to have larger families, with 41.5% reporting seven or more siblings, while Shokoh participants are more likely to have three or fewer siblings (52.5%). Tooth-brushing habits are high across both groups, with 91.6%. Shokoh participants exhibit slightly higher adherence (95%) compared to Afghan Turk (88.5%). Once-daily brushing is the most common practice, especially among Afghan Turk participants (82.5%), although 20.1% of participants across both groups report brushing twice daily ( Table 1).

**Table 1.** Socio-demographic characteristics of study participants by school.

	Afghan Turk		Shokoh		Total	
	n	%	n	%	n	%
Sex						
Boys	200	100.0	160	88.4	360	94.5
Girls	0	0.0	21	11.6	21	5.5
Age years						
12 or younger	0	0.0	75	41.4	75	19.7
13 to 15	97	48.5	71	39.2	168	44.1
16 or older	103	51.5	35	19.3	138	36.2
Maternal literacy						
Literate	74	37.0	85	47.0	159	41.7
Illiterate	126	63.0	96	53.0	222	58.3
Paternal literacy						
Literate	162	81.0	148	81.8	310	81.4
Illiterate	38	19.0	33	18.2	71	18.6
Maternal working status						
Housewife	182	91.0	150	82.9	332	87.1
Working	18	9.0	31	17.7	49	12.9
Paternal working status						
Jobs requiring education	78	39.0	66	36.5	144	37.8
Business	63	31.5	70	38.7	133	34.9
Jobs not requiring education	59	29.5	45	24.9	104	27.3
Number of siblings						
3 or less	29	14.5	95	52.5	124	32.5
4 to 6	88	44.0	63	34.8	151	39.6
7 or more	83	41.5	23	12.7	106	27.8
Tooth Brushing Habits						
Brushing	177	88.5	172	95.0	349	91.6
Not Brushing	23	11.5	9	5.0	32	8.4
Brushing frequency						
Once a Day	146	82.5	127	73.8	273	78.2
Twice a Day	26	14.7	44	25.6	70	20.1
Three Times a Day	5	2.8	1	0.6	6	1.7
Dental cleaning tools						
Use dental floss	64	31.5	60	33.1	123	32.3
Do not use dental floss	129	64.5	121	66.9	250	65.6
Use dental picks	8	4.0	0	0.0	8	2.1
Class						
5 to 7	0	0.0	108	59.7	108	28.3
8 to 9	90	45.0	36	19.9	126	33.1
10 to 12	110	55.0	37	20.4	147	38.6
Total	200	100.0	181	100.0	381	100.0

Table 2 presents a comparison of dental health metrics between Afghan Turk and Shokoh students. Over 10% of participants had at least one decayed deciduous tooth, with a higher prevalence among Shokoh students (22.1%) (p < 0.001), likely influenced by the younger age group in this school. In terms of permanent teeth, 56% of Afghan Turk students had at least one decayed tooth, compared to 87.3% in Shokoh. Afghan Turk and Shokoh students with 1-2 decayed permanent teeth comprised 35.5% and 40.3% of their groups, respectively. Moreover, 26.5% of Shokoh students had 3-4 decayed permanent teeth, compared to 13.0% in Afghan Turk, and the highest decay rate (5+ decayed teeth) affected 7.5% of Afghan Turk students and 10.5% of Shokoh students (p < 0.001). The rate of missing teeth also varied, with 91.0% of Afghan Turk students had no missing teeth, compared to 76.2% in Shokoh.

**Table 2.** Distribution of dental problems among students by school in Kabul city.

	Afghan Turk		Shokoh		Total	
	n	%	n	%	n	%
Number of decayed deciduous teeth					p value < 0.001	
Zero	196	98.0	141	77.9	337	88.5
1 to 2	4	2.0	30	16.6	34	8.9
3 or more	0	0.0	10	5.5	10	2.6
Number of decayed permanent teeth					p value < 0.001	
Zero	88	44.0	41	22.7	129	33.9
1 to 2	71	35.5	73	40.3	144	37.8
3 to 4	26	13.0	48	26.5	74	19.4
5 or more	15	7.5	19	10.5	34	8.9
Number of missing teeth					p value < 0.001	
Zero	182	91.0	138	76.2	320	84.0
1 to 2	15	7.5	35	19.3	50	13.1
3 or more	3	1.5	8	4.4	11	2.9
Oral hygiene					p value < 0.001	
Good	76	38.0	97	53.6	173	45.4
Average	111	55.5	54	29.8	165	43.3
Poor	13	6.5	30	16.6	43	11.3
Dental calculus					p value = 0.928	
Yes	93	46.5	85	47.0	178	46.7
No	107	53.5	96	53.0	203	53.3
Overall decayed teeth					p value < 0.001	
Yes	115	57.5	152	84.0	267	70.1
No	85	42.5	29	16.0	114	29.9

Good oral hygiene was more prevalent among Shokoh students than Afghan Turk students (53.6% vs. 38.0%). However, poor oral hygiene was also observed more frequently among Shokoh students (16.6%) compared with Afghan Turk students (6.5%) (p < 0.001). Rates of dental calculus were similar, affecting about 47% of students in both groups. However, overall decay presence was significantly higher in Shokoh, with 84.0% of students showing at least one decayed tooth, compared to 57.5% in Afghan Turk, highlighting a substantial difference in decay prevalence (p < 0.001).

Table 3 presents the mean Decayed, Missing, and Filled Teeth (DMFT) scores for students from Afghan Turk and Shokoh schools, categorized by sex and age groups. For Afghan Turk students, the overall mean DMFT score is 1.82 ± 2.17, with specific scores of 1.56 ± 1.97 for those aged 13-15 years and 2.06 ± 2.33 for students aged 16 years or older (p = 0.210). In contrast, Shokoh students have a mean DMFT score of 3.1 ± 2.64 for boys and 2.71 ± 2.03 for girls (p = 0.255). Additionally, Shokoh students exhibit a mean DMFT score of 2.45 ± 2.07 for the younger group (12 years or younger), 3.06 ± 2.25 for those in the 13-15 age range, and 4.34 ± 3.56 for the older group (16 years or older) (p = 0.010). The overall mean DMFT score for Shokoh students is 3.06 ± 2.57. The total mean DMFT score across both schools was 2.40 ± 2.45, with a highly significant p-value of <0.001, indicating substantial differences in dental health among the various groups studied.

**Table 3.** Distribution of DMFTs by age, sex and school.

	Afghan Turk			Shokoh			Overall		
	Mean	SD	P value	Mean	SD	P value	Mean	SD	P value
Sex									
Boy	1.82	2.17		3.1	2.64	0.689	2.39	2.47	0.255
Girl				2.71	2.03		2.71	2.03	
Age									
≤12 years				2.45	2.07	0.010	2.45	2.07	0.433
13-15 years	1.56	1.97	0.210	3.06	2.25		2.19	2.22	
≥16 years	2.06	2.33		4.34	3.56		2.64	2.86	
Overall	1.82	2.17		3.06	2.57		2.40	2.45	<0.001

The overall prevalence of dental caries among participants was 70.1%, with a significant gender difference: 68.9% of boys were affected compared to 90.5% of girls (p = 0.036). Age also significantly influenced caries prevalence, with 85.3% of children aged 12 years or younger exhibiting caries, compared to 68.5% in the 13-15 age group and 63.8% among those aged 16 years or older (p = 0.004). While there was a higher prevalence of caries among children of illiterate mothers (72.5%) compared to those with literate mothers (66.7%), this difference was not statistically significant (p = 0.218). Similarly, maternal working status did not show a significant impact on caries prevalence (p = 0.655) ( Table 4).

**Table 4.** Distribution of dental caries by socio-demographic characteristics and oral hygiene practices among students by school in Kabul city.

	Dental caries			
	Yes		No	
	n	%	n	%
Sex	p value = 0.036			
Boys	248	68.9	112	31.1
Girls	19	90.5	2	9.5
Age (years)	p value = 0.004			
≤12 years	64	85.3	11	14.7
13-15 years	115	68.5	53	31.5
≥16 years	88	63.8	50	36.2
Maternal literacy	p value = 0.218			
Literate	106	66.7	53	33.3
Illiterate	161	72.5	61	27.5
Paternal literacy	p value = 0.828			
Literate	218	70.3	99	29.7
Illiterate	49	69.0	22	31.0
Maternal working status	p value = 0.655			
Housewife	234	70.5	98	29.5
Working	33	67.3	16	32.7
Paternal working status	p value = 0.429			
Jobs requiring education	99	68.8	45	31.2
Business	90	67.7	43	32.3
Jobs not requiring education	78	75.0	26	25.0
Number of siblings	p value < 0.001			
3 or less	104	83.9	20	16.1
4 to 6	99	65.6	52	34.4
7 or more	64	60.4	42	39.6
Tooth Brushing Habits	p value = 0.025			
Brushing	239	68.5	110	31.5
Not Brushing	28	87.5	4	12.5
Brushing frequency	p value = 0.008			
Once a Day	198	72.5	75	27.5
Twice a Day	38	54.3	32	45.7
Three Times a Day	3	50.0	3	50.0
Dental cleaning tools	p value = 0.744			
Use dental floss	89	72.4	34	27.6
Do not use dental floss	172	68.8	78	31.2
Use dental picks	6	75.0	2	25.0
Oral hygiene	p value = 0.087			
Good	115	66.5	58	33.5
Average	116	70.3	49	29.7
Poor	36	83.7	7	16.3
Class	p value = 0.004			
5 to 7	89	82.4	19	17.6
8 to 9	83	65.9	43	34.1
10 to 12	95	64.6	52	35.4
Total	269	70.1	114	29.9

Interestingly, the number of siblings had an inverse correlation with caries prevalence: 83.9% of participants with three or fewer siblings experienced caries, while the prevalence decreased to 65.6% among those with four to six siblings and only 60.4% for those with seven or more siblings (p < 0.001). Tooth brushing habits were also associated with caries rates; 87.5% of participants who did not brush their teeth reported caries, in contrast to 68.5% of those who brushed regularly (p = 0.025). Furthermore, brushing frequency affected dental health outcomes, with 72.5% of participants brushing once daily exhibiting caries, compared to 54.3% of those brushing twice daily and 50% of those brushing three times a day (p = 0.008) ( Table 4).

Oral hygiene status was nearly significant, with 83.7% of students with poor oral hygiene experiencing caries compared to 66.5% of those with good hygiene (p = 0.087). Finally, class level significantly impacted caries rates: 82.4% of students in classes 5 to 7 reported caries, while lower rates of 65.9% and 64.6% were observed in classes 8 to 9 and 10 to 12, respectively (p = 0.004). Overall, these findings indicate that both demographic factors and oral health behaviors significantly contribute to the prevalence of dental caries among students ( Table 4).

The overall prevalence of dental calculus among participants was 46.7%, with no significant difference observed between the two schools (p = 0.928). However, age was a significant factor influencing the presence of dental calculus (p < 0.001). Among children aged 12 years or younger, 40.0% exhibited dental calculus, while the prevalence was 39.3% for those aged 13-15 years. This figure increased significantly to 59.4% for participants aged 16 years or older. Tooth brushing habits also significantly affected the prevalence of dental calculus; 43.8% of students who brushed their teeth were found to have calculus, in contrast to 78.1% of those who did not engage in regular brushing (p < 0.001). Additionally, the type of cleansing agent used was correlated with the presence of dental calculus (p < 0.001): only 31.1% of participants who used both toothpaste and mouthwash had calculus, while this figure rose to 45.7% among those who used only toothpaste and reached 75.0% for those who used no cleansing agents at all ( Table 5).

**Table 5.** Distribution of dental calculus and oral hygiene status by age and school among study participants.

	Dental calculus				Oral hygiene status					
	Yes		No		Good		Average		Poor	
	n	%	n	%	n	%	n	%	n	%
School	p value = 0.928				p value < 0.001					
Afghan Turk	93	46.5	107	53.5	76	38.0	111	55.5	13	6.5
Shokoh	85	47.0	96	53.0	97	53.6	54	29.8	30	16.6
Age	p value < 0.001				p value = 0.005					
≤12 years	30	40.0	45	60.0	45	60.0	22	29.3	8	10.7
13-15 years	66	39.3	102	60.7	81	48.2	70	41.7	17	10.1
≥16 years	82	59.4	56	40.6	47	34.1	73	52.9	18	13.0
Tooth Brushing Habits	p value < 0.001				p value < 0.001					
Brushing	153	43.8	196	56.2	171	49.0	143	41.0	35	10.0
Not Brushing	25	78.1	7	21.9	2	6.3	22	68.8	8	25.0
Cleansing agent	p value < 0.001				p value < 0.001					
Toothpaste & Mouthwash	14	31.1	31	68.9	29	64.4	12	26.7	4	8.9
Toothpaste	137	45.7	163	54.3	142	47.3	127	42.3	31	10.3
None	27	75.0	9	25.0	2	5.6	26	72.2	8	22.2
Total	178	46.7	203	53.3	173	45.4	165	43.3	43	11.3

Regarding oral hygiene status, a significant difference was observed between the two schools (p < 0.001). At Afghan Turk, only 38.0% of students were classified as having good oral hygiene, compared to a more favorable 53.6% in Shokoh. Age was also a determining factor for oral hygiene, with significant variations noted (p = 0.005). Among participants aged 12 years or younger, 60.0% maintained good oral hygiene, whereas this figure dropped to 48.2% for those aged 13-15 years and decreased further to just 34.1% for students aged 16 years or older. Tooth brushing habits were closely linked to oral hygiene status, with 49.0% of those who brushed their teeth reporting good hygiene, compared to a mere 6.3% of non-brushing students (p < 0.001). Furthermore, the type of cleansing agent used significantly correlated with oral hygiene status (p < 0.001). Among participants who utilized both toothpaste and mouthwash, 64.4% achieved good oral hygiene, while 47.3% of those using toothpaste only did, and only 5.6% of those using no cleansing agents reached the same standard. Overall, 45.4% of participants exhibited good oral hygiene, highlighting the urgent need for improved oral health education and practices among students ( Table 5).

## Discussion

This study presents a detailed analysis of dental health metrics among students from Afghan Turk and Shokoh schools, revealing significant differences in dental caries prevalence, oral hygiene status, and calculus presence. The high overall prevalence of dental caries (70.1%) and an especially high rate in Shokoh (84.0%) indicate a pressing public health concern. Contributing factors may include the younger age profile at Shokoh, where a substantial proportion of students aged 12 years or younger exhibited higher decay rates (85.3%). This trend aligns with existing literature, ^7– 9^ which suggests younger children face a greater risk for dental caries, often due to limited oral hygiene practices and diets high in sugar.

Previous research on schoolchildren aged 7-13 in Kabul reported an overall caries prevalence of 78.8%, ^6^ with differences in age distribution potentially explaining some variation between studies. Similar findings appear in a study by Bezian et al. in the Philippines, where 82.3% of students aged 11-13 had dental caries. ^10^ Studies from Iran by Hamissi et al. and from Poland by Milona et al. found prevalence rates of 75.5% and 88.6%, respectively, among adolescents. ^11, 12^ In Egypt, Marwa et al. reported a prevalence of 74.0% in children aged 3 to 18, ^7^ while another Egyptian study found a 53.1% prevalence among those aged 6 to 15. ^13^ In India, studies indicated prevalence rates of 68.8% among children aged 6-14 ^14^ and 61.4% among adolescents aged 12-15. ^15^

In Pakistan, a study reported a dental caries prevalence of 72.4%, ^16^ while another showed a 67.2% prevalence among children aged 5-14. ^17^ In Saudi Arabia, Al Zahrani et al. observed a prevalence of 74.6% among students aged 12 to 15, ^18^ with a related study noting 71.3% in those aged 10-13. ^19^ In contrast, a study in Albania reported a lower prevalence of 42.3% among students aged 7 to 15. ^20^ Similarly, in countries such as China, ^21^ Spain, ^22^ Croatia, ^23^ Portugal, ^24^ Brazil, ^25^ and Australia, ^26^ caries prevalence in comparable age groups was below 50%. A systematic review and meta-analysis by Kale identified a 61.0% (95% CI: 50.0–72.0) prevalence for children aged 12 and a 66.0% (95% CI: 59.0%-73.0%) prevalence for 15-year-olds in the Eastern Mediterranean Region. ^27^

Notably, 87.5% of students who did not brush regularly experienced caries, compared to 68.5% of those who brushed consistently, aligning with previous studies. ^28– 30^ This highlights the critical role of promoting effective oral hygiene practices, as brushing frequency significantly impacts dental health outcomes. Participants who brushed only once daily showed a caries prevalence of 72.5%, underscoring the need for education on optimal brushing practices, particularly the benefits of brushing twice daily. Interestingly, there was an inverse correlation between the number of siblings and caries prevalence; as the number of siblings increased, caries rates decreased. This may reflect shared family practices or resources that support better oral health in larger families, though a study in Taiwan observed a positive correlation between siblings and dental issues. ^31^ This discrepancy suggests further research is warranted to better understand the role of family dynamics in oral health behaviors.

Oral hygiene status also differed significantly between the two schools, with Shokoh students showing poorer hygiene (16.6%) compared to Afghan Turk students (6.5%). This disparity may stem from differences in access to dental care, nutritional choices, and health education within each community. Afghan Turk School, as a residential institution, provides consistent food and healthcare services to all students, which may contribute to the observed differences.

Finally, the study observed similar rates of dental calculus prevalence across both schools (46.7%), consistent with existing literature. ^32, 33^ However, age was a significant factor, with 59.4% of participants aged 16 or older showing calculus, suggesting that age-specific interventions may be beneficial. Proper use of oral hygiene products also showed positive effects; only 31.1% of students using both toothpaste and mouthwash had calculus, underscoring the value of effective oral care products in maintaining oral health.

The findings of this study also have important implications in relation to the WHO Global Oral Health Action Plan (GOHAP) 2023–2030, ^34^ which emphasizes the integration of oral health into public health systems and aims to achieve universal access to essential oral healthcare services. The high prevalence of dental caries and suboptimal oral hygiene observed among students in this study highlight the need for school-based oral health promotion programs, preventive interventions, and improved access to affordable dental care in Afghanistan. Strengthening oral health education, encouraging regular tooth brushing with fluoridated toothpaste, and expanding preventive dental services may contribute to reducing the burden of oral diseases among adolescents and support the goals of the WHO oral health strategy.

This study represents the first examination of dental caries prevalence among private school students in Kabul, offering important insights into their oral health status. Conducted by experienced dentists, the findings are highly reliable. By comparing two types of private schools, the study sheds light on the influence of educational environments on dental health. However, the limited sample of schools and geographic scope may affect the generalizability of the results. In addition, the limited inclusion of female participants, largely due to restrictions on girls’ school attendance beyond grade 6 in Afghanistan during the study period, may further limit the applicability of the findings to the broader adolescent population.

## Conclusions

In conclusion, this study highlights the high prevalence of dental caries and poor oral hygiene among students in Kabul City, emphasizing the urgent need for improved oral health practices within this population. While disparities were observed between Afghan Turk and Shokoh students, the overall findings point to critical determinants such as age, tooth brushing habits, and the use of effective oral hygiene products. These results underscore the importance of implementing school-based oral health programs, promoting regular dental care, and increasing awareness of proper oral hygiene practices. Collaborative efforts among policymakers, educators, and health professionals are essential to reduce dental disease prevalence and foster a healthier future generation across Afghanistan.

## Data Availability

The dataset supporting the findings of this study is available at the Figsahre: Dental Caries Study_AfghanTurk. https://doi.org/10.6084/m9.figshare.30771269 ^35^ The English version of the questionnaire used for data collection in this study is publicly available in Figshare: Questionnaire of the study Dataset. https://doi.org/10.6084/m9.figshare.31064665 ^36^ Data are available under the terms of the Creative Commons Attribution 4.0 International license (CC-BY 4.0).
