# Supplementary material for: Assessment of Dental Caries among Adolescents in Private Educational Institutions of Kabul City
Source: F1000Res. 2026 Jul 28;15:130. Originally published 2026 Jan 28. [Version 2] doi: 10.12688/f1000research.174734.2 (PMC13560052; doi:10.12688/f1000research.174734.2)
Supplement: Peer review history

# Reviewer report: Reviewer response for version 2

**DOI:** 10.5256/f1000research.203075.r505865

**Published:** 2026-08-14

**Authors:** Manuel S Thomas

**Affiliations:** 1 Manipal College of Dental Sciences, Mangalore, Karnataka, India

The authors have satisfactorily responded to all the queries and addressed the corrections and suggestions raised. The manuscript is acceptable from my end.

 Thanks and regards.

Is the work clearly and accurately presented and does it cite the current literature?

Partly

If applicable, is the statistical analysis and its interpretation appropriate?

Partly

Are all the source data underlying the results available to ensure full reproducibility?

Yes

Is the study design appropriate and is the work technically sound?

Partly

Are the conclusions drawn adequately supported by the results?

Yes

Are sufficient details of methods and analysis provided to allow replication by others?

Partly

Reviewer Expertise:

Dental caries

I confirm that I have read this submission and believe that I have an appropriate level of expertise to confirm that it is of an acceptable scientific standard.

# Reviewer report: Reviewer response for version 2

**DOI:** 10.5256/f1000research.203075.r505866

**Published:** 2026-08-05

**Authors:** Shahab Uddin Ahmadi

**Affiliations:** 1 Khatam-Al-Nabieen University,, Kabul,, Afghanistan

*I have carefully reviewed the revised version of the manuscript. The authors have adequately addressed the comments raised in my previous review, and I consider the revisions to be appropriate. Accordingly, I am pleased to recommend approval of the revised manuscript. I have no further comments and believe the manuscript is suitable for indexing.*

Is the work clearly and accurately presented and does it cite the current literature?

Partly

If applicable, is the statistical analysis and its interpretation appropriate?

Partly

Are all the source data underlying the results available to ensure full reproducibility?

Partly

Is the study design appropriate and is the work technically sound?

Partly

Are the conclusions drawn adequately supported by the results?

Partly

Are sufficient details of methods and analysis provided to allow replication by others?

Partly

Reviewer Expertise:

NA

I confirm that I have read this submission and believe that I have an appropriate level of expertise to confirm that it is of an acceptable scientific standard.

# Reviewer report: Reviewer response for version 1

**DOI:** 10.5256/f1000research.192658.r482309

**Published:** 2026-05-26

**Authors:** Shahab Uddin Ahmadi

**Affiliations:** 1 Khatam-Al-Nabieen University,, Kabul,, Afghanistan

This manuscript addresses an important and underreported public health issue by evaluating the prevalence of dental caries among adolescents attending private schools in Kabul City. The study contributes valuable epidemiological data from Afghanistan, where oral health research remains limited. The manuscript is generally well organized and supported by relevant regional and international literature. The inclusion of DMFT scores and oral hygiene variables strengthens the public health relevance of the study.

 However, several issues should be addressed before the manuscript can be considered scientifically robust.

 Major concerns include inconsistencies in statistical reporting and interpretation. The manuscript states that p < 0.005 was considered statistically significant, whereas the analyses throughout the paper appear to follow the conventional p < 0.05 threshold. Additionally, the reported mean age standard deviation (14.6 ± 12.13 years) appears implausible for a population aged 10–18 years and should be verified.

 There are also interpretation inconsistencies in the Results section. For example, the text incorrectly states that nearly two-thirds of Afghan Turk participants had literate mothers, whereas the table indicates the opposite. Similarly, the oral hygiene interpretation between schools contradicts the reported table values. These errors should be carefully corrected.

 Methodologically, the study would benefit from clearer explanation of oral hygiene assessment criteria and examiner calibration procedures. No information is provided regarding inter-examiner reliability or use of standardized oral hygiene indices. The sampling method also appears to be convenience-based and should be explicitly acknowledged as a limitation.

 The statistical methods are generally appropriate for a cross-sectional study; however, causal wording such as “influenced” or “affected” should be replaced with more appropriate associative language. Table 3 also requires reformatting for clarity.

 The discussion section effectively compares findings with previous regional and international studies, although greater analytical interpretation of the observed differences between schools would improve the manuscript further. Additionally, one cited reference appears unrelated to caries prevalence and should be verified.

 Overall, this study has good potential and addresses an important public health topic. With moderate revision, correction of inconsistencies, clarification of methods, and language polishing, the manuscript could make a valuable contribution to oral health literature in Afghanistan.

Is the work clearly and accurately presented and does it cite the current literature?

Partly

If applicable, is the statistical analysis and its interpretation appropriate?

Partly

Are all the source data underlying the results available to ensure full reproducibility?

Partly

Is the study design appropriate and is the work technically sound?

Partly

Are the conclusions drawn adequately supported by the results?

Partly

Are sufficient details of methods and analysis provided to allow replication by others?

Partly

Reviewer Expertise:

Dentistry, Dental Public Health, Oral Epidemiology, Preventive Dentistry, Community Oral Health

I confirm that I have read this submission and believe that I have an appropriate level of expertise to confirm that it is of an acceptable scientific standard, however I have significant reservations, as outlined above.

# Response

**Published:** 2026-06-02

**Authors:** Ali Maisam Eshraqi

Approved With Reservations

 This manuscript addresses an important and underreported public health issue by evaluating the prevalence of dental caries among adolescents attending private schools in Kabul City. The study contributes valuable epidemiological data from Afghanistan, where oral health research remains limited. The manuscript is generally well organized and supported by relevant regional and international literature. The inclusion of DMFT scores and oral hygiene variables strengthens the public health relevance of the study.

 However, several issues should be addressed before the manuscript can be considered scientifically robust.

 Major concerns include inconsistencies in statistical reporting and interpretation. The manuscript states that p < 0.005 was considered statistically significant, whereas the analyses throughout the paper appear to follow the conventional p < 0.05 threshold. Additionally, the reported mean age standard deviation (14.6 ± 12.13 years) appears implausible for a population aged 10–18 years and should be verified.

**Thanks, the p-value<0.05 was considered statistically significant. Apologies for the typo error there was one extra zero we have corrected it.**

**The standard deviation was also a typo error. It is 2.13 and I have corrected it.**

 There are also interpretation inconsistencies in the Results section. For example, the text incorrectly states that nearly two-thirds of Afghan Turk participants had literate mothers, whereas the table indicates the opposite. Similarly, the oral hygiene interpretation between schools contradicts the reported table values. These errors should be carefully corrected.

**Thanks, we have corrected the errors.**

 Methodologically, the study would benefit from clearer explanation of oral hygiene assessment criteria and examiner calibration procedures. No information is provided regarding inter-examiner reliability or use of standardized oral hygiene indices. The sampling method also appears to be convenience-based and should be explicitly acknowledged as a limitation.

**We have added details regarding the sampling method. The definition of oral hygiene used in this study was already provided in the Methods section. All oral examinations were performed by an experienced dentist, while other members of the research team assisted with data collection.**

 The statistical methods are generally appropriate for a cross-sectional study; however, causal wording such as “influenced” or “affected” should be replaced with more appropriate associative language. Table 3 also requires reformatting for clarity.

**Thank you for your comment. We agree that causal inferences cannot be made from a cross-sectional study. However, our manuscript uses associative language and does not imply causality. As a cross-sectional study, our objective was to identify factors associated with oral hygiene status. We have also checked and reformatted any incorrect wording in Table 3 to improve clarity.**

 The discussion section effectively compares findings with previous regional and international studies, although greater analytical interpretation of the observed differences between schools would improve the manuscript further. Additionally, one cited reference appears unrelated to caries prevalence and should be verified.

**Thanks for noticing that. We have replaced it with the correct one. The same author but in different study.**

 Overall, this study has good potential and addresses an important public health topic. With moderate revision, correction of inconsistencies, clarification of methods, and language polishing, the manuscript could make a valuable contribution to oral health literature in Afghanistan.

# Reviewer report: Reviewer response for version 1

**DOI:** 10.5256/f1000research.192658.r482308

**Published:** 2026-05-20

**Authors:** Manuel S Thomas

**Affiliations:** 1 Manipal College of Dental Sciences, Mangalore, Karnataka, India

- Since epidemiological data from Afghanistan is limited, this study can add value to future oral health preventive planning and school-based interventions; however, there are certain concerns.
- The introduction does not adequately justify the choice of adolescents or private schools in Kabul, and the authors should clearly explain these selections to strengthen the study rationale.
- The reported mean age (14.6 ± 12.13 years) is incorrect, as the standard deviation is not possible within the 10–18 year range; the authors should verify and correct the age statistics and review all descriptive data for errors.
- The manuscript inconsistently interprets statistical significance, as results with p-values between 0.005 and 0.05 are reported as significant despite the stated threshold; the authors should clarify the correct cutoff (likely p < 0.05) and revise the manuscript accordingly.
- The manuscript does not provide enough detail on participant recruitment and sampling, so the authors should clarify the sampling method, eligible population, participation rates, and any exclusion criteria to assess representativeness and bias.
- The manuscript does not clearly define dental caries, as it is unclear whether non‑cavitated lesions and also why wasn’t filled teeth were included; the authors should clarify these criteria.
- The text and table are inconsistent, as the reported maternal literacy percentages appear reversed; the authors should correct all discrepancies between the text and tables.
- Reference 18 is unrelated to dental caries prevalence and should be replaced with an appropriate epidemiological citation.
- Multiple grammatical and typographical errors require language editing. For example, “Dental carries” should be corrected to “Dental caries.” There are several spacing and punctuation inconsistencies throughout the manuscript.
- The manuscript can include the limited inclusion of female participants and its effect on generalizability.
- The discussion can interpretation of the findings and relate them to the WHO Global Oral Health Action Plan (GOHAP) 2023–2030, which aims to achieve universal access to oral healthcare.

Is the work clearly and accurately presented and does it cite the current literature?

Partly

If applicable, is the statistical analysis and its interpretation appropriate?

Partly

Are all the source data underlying the results available to ensure full reproducibility?

Yes

Is the study design appropriate and is the work technically sound?

Partly

Are the conclusions drawn adequately supported by the results?

Yes

Are sufficient details of methods and analysis provided to allow replication by others?

Partly

Reviewer Expertise:

Dental caries

I confirm that I have read this submission and believe that I have an appropriate level of expertise to confirm that it is of an acceptable scientific standard, however I have significant reservations, as outlined above.

# Response

**Published:** 2026-06-02

**Authors:** Ali Maisam Eshraqi

Reviewer 1

 Approved With Reservations

     Since epidemiological data from Afghanistan is limited, this study can add value to future oral health preventive planning and school-based interventions; however, there are certain concerns.

     The introduction does not adequately justify the choice of adolescents or private schools in Kabul, and the authors should clearly explain these selections to strengthen the study rationale.

 Thanks, we have updated the rational of the study.

     The reported mean age (14.6 ± 12.13 years) is incorrect, as the standard deviation is not possible within the 10–18 year range; the authors should verify and correct the age statistics and review all descriptive data for errors.

 Thanks for noticing that. It was a typo error. It is 2.13 and I have corrected it and reviewed the descriptive data.

     The manuscript inconsistently interprets statistical significance, as results with p-values between 0.005 and 0.05 are reported as significant despite the stated threshold; the authors should clarify the correct cutoff (likely p < 0.05) and revise the manuscript accordingly.

 Thanks, the p-value<0.05 was considered statistically significant. Again, apologies for the typo there was one extra zero we have corrected it.

     The manuscript does not provide enough detail on participant recruitment and sampling, so the authors should clarify the sampling method, eligible population, participation rates, and any exclusion criteria to assess representativeness and bias.

 We have added details of sampling methods, including inclusion and inclusion criteria. Regarding participation rate all of the students in the selected classes participated in the survey.

     The manuscript does not clearly define dental caries, as it is unclear whether non‑cavitated lesions and also why wasn’t filled teeth were included; the authors should clarify these criteria.

 We have updated the definition of dental caries in this study and explained the reason of why filled teeth included in the DMFT.

     The text and table are inconsistent, as the reported maternal literacy percentages appear reversed; the authors should correct all discrepancies between the text and tables.

 Thanks, we have corrected the errors.

     Reference 18 is unrelated to dental caries prevalence and should be replaced with an appropriate epidemiological citation.

 Thanks for noticing that. We have replaced it with the correct one. The same author but in different study.

     Multiple grammatical and typographical errors require language editing. For example, “Dental carries” should be corrected to “Dental caries.” There are several spacing and punctuation inconsistencies throughout the manuscript.

 Thanks for your comment. Unfortunately, the title of a study has been written with that error as we used the endnote software, we haven’t noticed that error. We have corrected this and other typographical errors.

     The manuscript can include the limited inclusion of female participants and its effect on generalizability.

 We have updated the limitations of the study.

     The discussion can interpretation of the findings and relate them to the WHO Global Oral Health Action Plan (GOHAP) 2023–2030, which aims to achieve universal access to oral healthcare.

 We have added a paragraph about this in the discussion.
